# Microbial Decontamination of Red Wine by Pulsed Electric Fields (PEF) after Alcoholic and Malolactic Fermentation: Effect on *Saccharomyces cerevisiae*, *Oenococcus oeni*, and Oenological Parameters during Storage

**DOI:** 10.3390/foods12020278

**Published:** 2023-01-06

**Authors:** Carlota Delso, Alejandro Berzosa, Jorge Sanz, Ignacio Álvarez, Javier Raso

**Affiliations:** Food Technology, Facultad de Veterinaria, Instituto Agroalimentario de Aragón-IA2, Universidad de Zaragoza-CITA, 50013 Zaragoza, Spain

**Keywords:** red wine, pulsed electric fields, microorganisms, inactivation, sulfur dioxide, shelf-life

## Abstract

New techniques are required to replace the use of sulfur dioxide (SO_2_) or of sterilizing filtration in wineries, due to those methods’ drawbacks. Pulsed electric fields (PEF) is a technology capable of inactivating microorganisms at low temperatures in a continuous flow with no detrimental effect on food properties. In the present study, PEF technology was evaluated for purposes of microbial decontamination of red wines after alcoholic and malolactic fermentation, respectively. PEF combined with SO_2_ was evaluated in terms of microbial stability and physicochemical parameters over a period of four months. Furthermore, the effect of PEF on the sensory properties of red wine was compared with the sterilizing filtration method. Results showed that up to 4.0 Log_10_ cycles of *S. cerevisiae* and *O. oeni* could be eradicated by PEF and sublethal damages and a synergetic effect with SO_2_ were also observed, respectively. After 4 months, wine treated by PEF after alcoholic fermentation was free of viable yeasts; and less than 100 CFU/mL of *O. oeni* cells were viable in PEF-treated wine added with 20 ppm of SO_2_ after malolactic fermentation. No detrimental qualities were found, neither in terms of oenological parameters, nor in the sensory parameters of wines subjected to PEF after storage time.

## 1. Introduction

Wine is a fermented beverage with particular characteristics, such as a low pH (3.0–3.9) and the presence of ethanol (8–16% *v*/*v*), which thereby restrain the proliferation of foodborne pathogenic microorganisms and spore-forming bacteria. However, certain groups of microorganisms are able to grow in wines and spoil them in different stages of the winemaking process [1]. Such spoilage microorganisms are usually either the endogenous microbiota of grape skins, or microorganisms stemming from contact surfaces and equipment in wineries [2]. On the other hand, microorganisms that are essential in wine production, such as *Saccharomyces cerevisiae* and lactic acid bacteria (LAB), responsible for alcoholic and malolactic fermentation, respectively, may likewise be involved in wine spoilage. These microorganisms therefore need to be controlled after fermentation to avoid undesired re-fermentation, which, in turn, may lead to off-flavors, increments in volatile acidity, or even the production of biogenic amines [3].

To obtain quality wines and avoid economic losses, it is essential to control all of the involved spoiling microorganisms in grapes, must, and wine, as well as on surfaces in wineries. Apart from rigorous standard cleaning and disinfection plans, the conventional method for microbial control in wineries is the application of sulfur dioxide (SO_2_) [4]. Sulfur dioxide has bacteriostatic and antifungal activity but also plays an essential role as an antioxidant: all of these functions make it a thoroughly convenient compound for wine preservation [5]. Sulfur dioxide is usually dosed along the production process: when grapes are received, after alcoholic and malolactic fermentation, and usually prior to bottling in order to ensure wine stability during distribution. When SO_2_ is incorporated into must or wine, a fraction of it will react with sugars, aldehydes, or ketones [6]. Consequently, two classes of sulfites are found in wine: free and bound. The free sulfites determine how much SO_2_ is available in its most active, molecular form to help protect the wine from oxidation and spoilage. The bound sulfites are those which have reacted with other molecules. Total sulfite concentration is the sum of the free and bound sulfites.

However, the widespread use of sulfur dioxide in winemaking is currently being called into question, due to its potential toxicity for human health, the sensitivity of certain people allergic to SO_2_, and an increasing overall consumer rejection of chemical preservatives [7]. Another procedure frequently conducted in wineries to guarantee complete microbial decontamination is sterilizing filtration prior to bottling. However, filters with a nominal pore size of 0.45 µm generally used in sterilizing filtration can be ineffective in retaining certain smaller-sized bacteria. Furthermore, wine microfiltration usually implies fouling, regeneration problems, and high operational costs [8]. Moreover, this procedure is frequently controversial in wineries due to its deleterious effect on the chemical and sensory characteristics of wine, especially red wine [9]. The industry is thus searching for suitable alternative methods for microbial control that do not modify the properties of wine. The capability of PEF for inactivating vegetative forms of microbial cells at lower temperatures than those used in thermal processing [10,11,12] may prove to be thoroughly useful for wineries as a physical procedure for microbial decontamination.

PEF technology consists in the application of short pulses (microseconds) of high voltages (kV) to a product located between two electrodes. The electric field thereby generated produces the electroporation of cells. Electroporation compromises the permeability of the cytoplasmic membrane of microorganisms, leading to the loss of microbial homeostasis and, ultimately, to cell death. The efficacy of PEF for microbial inactivation, together with the prospect of applying it at high rates of continuous flow thanks to the availability of flexible commercial devices on the market, makes this technology a reasonable potential method for liquid food decontamination [13]. Several studies have demonstrated the effectivity of PEF in inactivating diverse microorganisms in acidic beverages, such as fruit juices, but with a noticeable variability in the amount of reported lethality [14,15,16,17]. Some research has been performed regarding the use of PEF for microbial inactivation in wine. Puértolas et al., (2009) [18] characterized the PEF resistance of some of the most common microorganisms involved in wine spoilage in batch conditions; González-Arenzana et al., (2015) [19] extended that approach to a greater number of microorganisms, performing the treatments in continuous flow. These studies demonstrated that PEF treatments at intensities over 20 kV/cm were effective for all of the microorganisms investigated (yeasts, lactic acid bacteria, and acetic acid bacteria) in the range of 3.0 to 4.0 Log_10_ cycles of inactivation. Further studies have demonstrated that PEF treatments do not cause detrimental effects: neither in terms of oenological parameters, nor on the sensory properties of wine, even after long-term storage [20,21]. However, all of these studies were conducted at high electric fields (which would be difficult to apply on an industrial scale with the current commercial PEF units) and/or without reporting the free SO_2_ concentration present in wines, which may exert an influence on the efficacy of PEF.

The objective of our study was to characterize the PEF resistance of *Saccharomyces cerevisiae* and *Oenococcus oeni* in red wine after alcoholic and malolactic fermentation, respectively. Once defined the most suitable PEF processing conditions, the impact of these treatments on the microbial population, and the oenological parameters of wines after 4 months of storage were investigated.

## 2. Material and Methods

### 2.1. Red Wine Samples

The Cooperative San Juan Bautista (Fuendejalón, Aragón, Spain) provided 50 L of *Grenache* wine immediately after they had undergone alcoholic fermentation (AF), or malolactic fermentation (MLF). Alcoholic fermentation was performed by the *S. cerevisiae var. bayanus* (CHP Levuline, OENOFRANCE, Magenta, France) strain, and malolactic fermentation by *Oenococcus oeni* (Viniferm OE AG-20, Agrovin, Ciudad Real, Spain). The initial oenological parameters of the two wines after AF and MLF are shown in Table 1. An aliquot of 10 L of each wine was used to characterize microbial resistance to PEF, and the effect of combining PEF with SO_2_. The rest of the wine samples were kept under refrigeration (4 °C) and no-light conditions in view of conducting an experiment on the impact of PEF on their microbial population, as well as on oenological parameters after AF and MLF during storage. Immediately after PEF treatments, SO_2_ was added, and wine samples were distributed in sterilized glass bottles of 500 mL and stored at 18 °C for 4 months. PEF-treated and untreated wines with and without sulfites were monitored in terms of their microbial population and chemical parameters over a period of 4 months.

### 2.2. PEF Processing

PEF treatments were applied in a continuous flow by means of a commercial generator (Vitave, Prague, Czech Republic) able to deliver pulses of up to 20 kV. Square waveform monopolar pulses were delivered in a parallel titanium electrode chamber with a 0.4 cm gap (3.0 × 0.5 cm). Wines tempered at 20 °C in a heat exchanger placed prior to the treatment chamber were pumped by a peristaltic pump (BVP, Ismatec, Wertheim, Germany) at different flow rates into the chamber. After the treatments, wines were cooled down in less than 5 s to under 20 °C in a second heat exchanger located after the treatment chamber. The actual voltage during treatments was measured by a high voltage probe (Tektronik, P6015A, Wilsonville, OR, USA) connected to an oscilloscope (Tektronik, TDS 220). Inlet and outlet temperatures were measured by a type K thermocouple inserted in the circuit (Ahlborn, Holzkirchen, Germany).

In preliminary studies, a matrix of different PEF parameters was tested with the aim of identifying optimal conditions for microbial decontamination. Wines after AF (1.9 mS/cm) and MLF (2.0 mS/cm) were pumped at 10 L/h, resulting in a residence time of 0.22 s in the chamber. Pulses of 10 µs were delivered at electric field strengths of 15, 20, and 25 kV/cm, and at a repetition rate ranging from 8.0 to 80 Hz, corresponding to effective treatment times ranging from 20 to 175 µs. These treatments corresponded to total specific energies of 35 to 120 kJ/kg, thereby implying an exit temperature in the range of 30 to 50 °C ± 2 °C. For the storage study, the two PEF treatments selected for each red wine were applied at 15 kV/cm. Wine after AF was treated with total specific energies of 39 and 97.2 kJ/kg (exit temperatures of 30 and 45 °C, respectively), whereas for wine after MLF, energies were 77.8 and 116.7 kJ/kg (exit temperatures of 40 and 50 °C, respectively).

### 2.3. Sulfite Addition

Immediately after PEF treatments, wine samples were added with the corresponding amount of SO_2_. A stock solution of 25 g/L of SO_2_ was prepared from potassium bisulfite (Sigma, Burlington, MA, USA). PEF-treated and untreated wines were dosed with 0, 20, and 30 ppm of SO_2_. The total free SO_2_ content at the starting point of the storage study was the sum of the initial free SO_2_ content in wine after AF (19 ppm) and after MLF (10 ppm) plus the corresponding doses of SO_2_ added in this step.

### 2.4. Microbial Analysis

Microbial survivors were measured by the corresponding plating of aliquots of wine samples diluted in peptone water (Oxoid, Basingtok, Hampshire, UK) and plated onto the appropriate agar medium. For yeast enumeration, Potato Dextroxe Agar (Oxoid) was used, and plates were incubated at 25 °C for 48 h. Lactic acid bacteria (LAB) survivors were enumerated in Mann Rogosa Sharpe (MRS) Agar (Oxoid) and the plates were incubated in anaerobic conditions (<1% O_2_) at 30 °C for 24 to 72 h. After the plate incubation, the number of counted colonies corresponds with the number of viable microorganisms expressed as a colony form unit per milliliter (CFU/mL) or its decimal logarithm (Log_10_ CFU/mL). The survival fraction was calculated by dividing the number of microorganisms that survived the treatment (*N_t_*) by the initial number of viable cells (*N*_0_).

### 2.5. Analysis of Oenological Parameters

The initial and final oenological parameters of all of the wine samples were measured. The pH, glucose-fructose, % ethanol, and total and volatile acidity were analyzed by FTIR spectroscopy using MIURA 200 and BACCHUS 3 MultiSpec models (TDI, Barcelona Spain). Absorbance measurements were performed after centrifuging wine samples in an Eppendorf AG centrifuge for 15 min at 3000 rpm (Eppendorf, Hamburg, Germany). All spectrophotometric determinations were measured by spectrophotometer (DS-11, DeNovix, Wilmington, DE, USA). The color index (CI) was determined as the sum of absorbance at 420, 520, and 620 nm. The total polyphenol index (TPI) was determined by measuring the direct absorbance at 280 nm. Total anthocyanins (TAC) expressed in malvidin-3-glucoside (mg/L) were calculated by determining the absorbance at 520 nm of samples diluted 1/10 (*v*/*v*) in 1% (*v*/*v*) of HCl, adapted from Maza et al., (2019) [22].

The determination of total and free sulfur dioxide (SO_2_) was performed by the Ripper method, which is based on an oxidation-reduction titration using iodine as a reagent in an acid medium in the presence of starch. Briefly, 1 mL of starch (1%) and 2 mL of sulfuric acid 1/3 *w*/*v* vinikit (PanReac, Barcelona, Spain) were added to 15 mL of wine. This solution was titrated with an iodine solution (0.01 N) until a blue color appeared.

### 2.6. Sensory Evaluation of PEF Treatments in a Commercial Wine

To evaluate the impact of PEF on sensory properties, independent experiments were performed in a finished red wine prior to being bottled. This red wine was treated by PEF at two different intensities and compared with the same wine after sterilizing filtration in the facilities of the winery. A total amount of 50 L of red wine (1.5 mS/cm) was processed by PEF at a flow rate of 25 L/h. Pulses of 5 µs were applied at 107 and 190 Hz at 15 kV/cm at the total specific energies of 77.8 of 155.6 kJ/kg that corresponded to exit temperatures of 40 and 60 ± 2.0 °C, respectively. The cooling exchanger placed immediately after the PEF chamber reduced the wine’s temperature to under 20 °C in less than 5 s before bottling. After 1 month of storage, physicochemical and sensory analyses were performed. Oenological parameters were measured as previously described (Section 2.5), and a sensory evaluation was carried out by a complete sensory triangle discrimination test, performed by 16 panelists from the Campo de Borja Appellation of Origin (nine men/six women ages 26 to 58). Panelists were distributed in individual booths and were given no information regarding the testing samples. Comparisons performed in the triangle test were between the sterilized filtered wine and the PEF-treated wines, using a completely randomized design. Samples were previously tempered to room temperature and 20 mL were served in clear wine glasses (ISO NORM 3591) [23]. Panelists had to distinguish the one different sample among the three samples presented in each batch, by taste and/or aroma.

### 2.7. Statistical Analysis

Samples were analyzed in three independent replicates and data are expressed as the mean ± the standard deviation. When called for, one-way analysis of variance (ANOVA) and Tukey tests using GraphPad Prism (Graph-Pad Software, San Diego, CA, USA) were performed to evaluate the significance of differences among the mean values. Differences were considered significant at *p* ≤ 0.05. The significant difference for the triangular test was determined using statistical tables reported by Roessler et al., (1948) [24].

## 3. Results and Discussion

### 3.1. PEF-Resistance of Saccharomyces cerevisiae and Oenococcus oeni

Preliminary experiments were conducted to determine the resistance of *S. cerevisiae* to PEF treatments of *Grenache* red wine after alcoholic fermentation, and of *O. oeni* after malolactic fermentation. The ultimate objective of these experiments was to select PEF processing conditions for subsequent study, aiming to evaluate the evolution of these microbial populations in each wine during storage.

**Figure 1:**
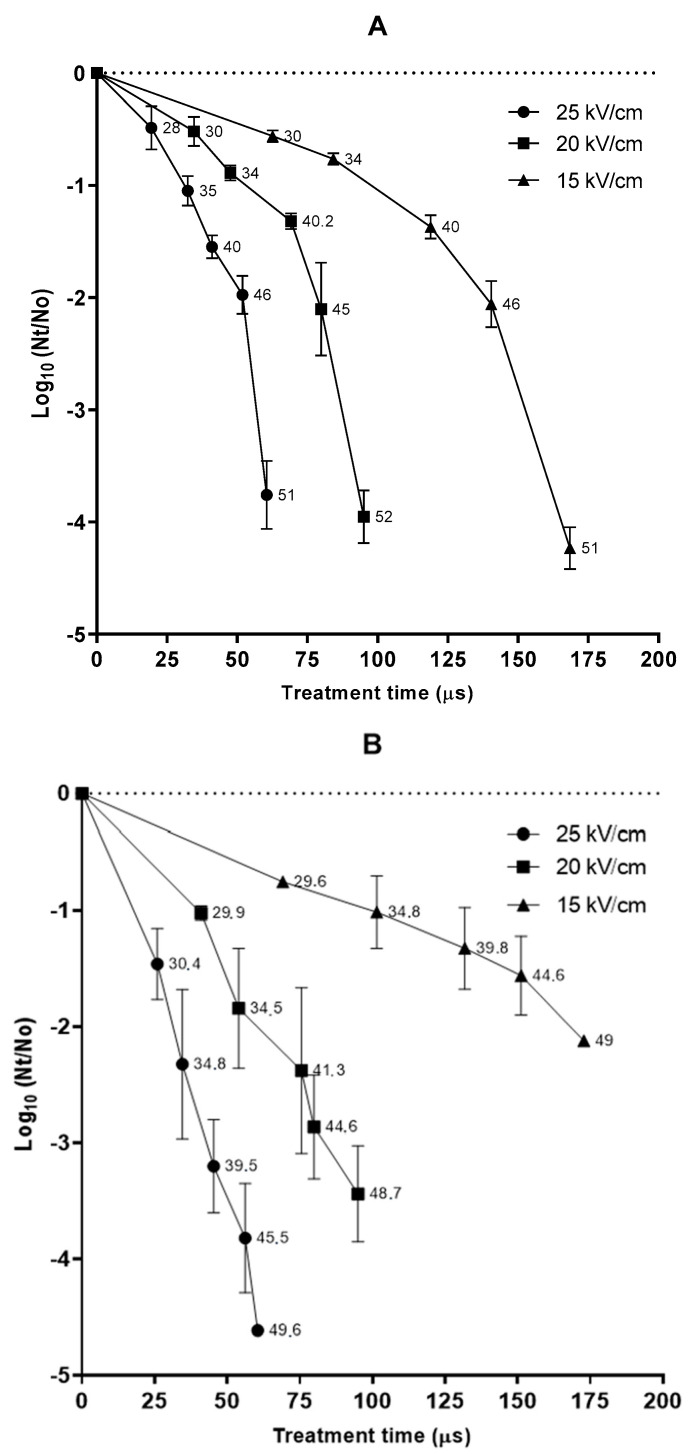
Survival curves in wine of *S. cerevisiae* (**A**) and *O. oeini* (**B**) after alcoholic (AF) and malolactic (MLF) fermentation, respectively, at different electric field strengths. Numbers near the dots indicate the outlet temperature achieved during treatments.

Survival curves corresponding to the inactivation of *S. cerevisiae* and *O. oeni* at different electric fields (15, 20, and 25 kV/cm) are shown in Figure 1A,B, respectively. The numbers next to the symbols indicate the outlet temperature of wine attained during PEF processing. Due to the wine’s short residence time in the treatment chamber (0.22 s), no heat is exchanged with the surroundings. Consequently, all of the electrical energy delivered to the treatment chamber to generate the electric field is transformed into heat, thereby increasing the wine’s temperature. However, in the experimental approach used in this study, wine was cooled below 20 °C within 5 s after the PEF treatment, independently of the outlet temperature. As can be observed in the two graphs, the temperature increased with the treatment time (number of pulses × pulse width).

Figure 1 shows that the inactivation kinetics of the two microorganisms is different. In the case of *S. cervisiae*, the survival curve’s shape is concave upwards, whereas in the case of *O. oeni* a linear behavior can be observed. The shape of the survival curves of *S. cerevisiae* could be explained by the effect of temperature on the membrane’s stability as a consequence of the phase transition of the phospholipids from gel to the liquid-crystalline phase. A sudden change in inactivation kinetics can be observed when the output temperature of the wine was over 40 °C. This change would indicate that above 40 °C the cytoplasmic membrane of *S. cerevisiae* is more vulnerable to the pore formation caused by PEF [25]. The positive effect of treatment medium temperature on microbial inactivation has been previously reported by several authors [10,26]. However, in the case of *O. oeni*, no radical changes in the inactivation kinetics were observed in the same range of outlet temperatures. This might indicate differences in the cytoplasmic membrane composition of both microorganisms, which could vary in terms of the manner in which the temperature affects the phase transition of the phospholipids.

The inactivation of the two microorganisms increased with the electric field, whereby shorter treatments at higher electric fields strengths were required to achieve a given level of inactivation. For example, to inactivate 2.0 Log_10_ cycles of the population of *S. cerevisiae*, the treatment time decreased from 140 to 52 µs when the electric field was increased from 15 to 25 kV/cm. The same electric field increment reduced the treatment time from 180 to 40 µs to achieve a similar inactivation in *O. oeni*. In the case of *S. cerevisiae*, the outlet temperature for both treatments applied at different electric field strengths was the same (45 °C), thereby indicating that the total specific energy of the treatments applied at different electric fields was equivalent (97 kJ/kg). Huang et al. (2014) and Puértolas et al. (2009) [18,27] also reported that the electric field did not modify PEF lethality on different *Saccharomyces* strains suspended in must or wine when treatments of the same specific energy were applied. However, in the case of *O. oeni*, the total specific energy required for achieving an equivalent lethality was lower when the electric field strength was increased. For example, in order to obtain 2.0 Log_10_ cycles, the total specific energy decreased from 117 to 49 kJ/kg when the electric field was increased from 15 to 25 kV/cm. Consequently, whereas the outlet temperature of the treatment applied at 15 kV/cm was around 50 °C, the outlet temperature of the treatment applied at 25 kV/cm lay in the range of 30.4 to 34.8 °C. The total specific energy of a PEF treatment depends on the applied voltage, total treatment time, and the electrical resistance of the treatment chamber. Since total specific energy is an integrate parameter that involves electric field strength and treatment time, it has been proposed as a single parameter to define the intensity of a PEF treatment [28]. This approach could be considered in the case of the strain of *S. cerevisiae* used in this study, upon which equivalent total specific energy delivered at different electric field strengths had the same lethal effect. However, in most microorganisms observed in the *O. oeni* strain, treatments of the same specific energy are more effective in terms of microbial inactivation when the applied electric field strength is higher [29]. Therefore, in this case, to define the intensity of a given PEF treatment, it is necessary to report both the electric field strength and total delivered specific energy.

It has generally been reported that yeasts are more sensitive to PEF than bacteria [30,31,32]. The high PEF sensitivity of yeast has been associated with the fact that larger cells require a lower critical electric field to achieve the transmembrane potential threshold for the manifestation of electroporation. However, other intrinsic microbial factors in addition to cell size seem to play a role in microbial resistance to PEF. Whereas at 15 kV/cm the resistance of the two microorganisms to PEF treatments of different duration was similar, at higher electric field strength *O. oeni* was slightly more sensitive than *S. cerevisiae*. For example, a treatment of 20 kV/cm for 70 µs that corresponded with an exit temperature of 40 °C inactivated around 1.5 Log_10_ cycles of *S. cerevisiae*, and around 2.5 Log_10_ of *O. oeni*. The higher resistance of *S. cerevisiae* to PEF might be explained by the fact that, in contrast to other studies in which the wine was contaminated with yeast previously grown in laboratory media, our investigation was conducted with the cells that had performed the alcoholic fermentation. The presence of ethanol during the growth of those cells could provoke changes in the composition or structure of their cytoplasmic membrane [33,34], which, in turn, could lead to a cross-protection against the electroporation brought about by PEF.

It is difficult to compare the PEF resistance of the strains used in this investigation with reported data in view of the widely varying protocols employed for PEF application, the differences among the investigated strains, their physiological state, and the variabilities in the composition of wines. The PEF resistance of yeast and lactic acid bacteria in wine has been reported in previous studies. Treatments at an electric field above 17 kV/cm and 90 kJ/kg were required to achieve between 2.0 and 3.0 Log_10_ cycles of the inactivation of the *Saccharomyces* population [18,19]. After alcoholic fermentation, intense PEF treatments (33 kV/cm; 158 kJ/kg; 105 µs) inactivated between 3.7 and 7.2 Log_10_ cycles of the population of yeast cells involved in the fermentation process [35]. Meanwhile, lethality reported for *O. oeni* ranged from 1.5 to 3.2 Log_10_ when PEF treatments of 17–23 kV/cm and 60–100 kJ/kg were applied [19,20].

### 3.2. Inactivation of Saccharomyces cerevisiae and Oenococcus oeni by Combining Moderate PEF Treatments with SO_2_

**Figure 2:**
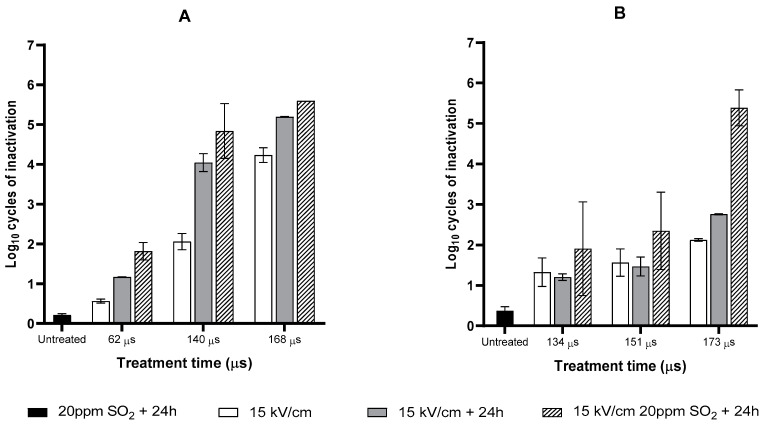
Comparison of the Log_10_ cycles of inactivation of *S. cerevisiae* (**A**) and *O. oeini* (**B**) after alcoholic (AF) and malolactic (MLF) fermentation, respectively, treated at 15 kV/cm and plated immediately after PEF treatment, or plated after 24 h of incubation with 0 or 20 ppm of SO_2_. PEF treatments 62 µs, 39 kJ/kg, exit temperature: 30 °C; 134 µs, 78 kJ/kg, exit temperature: 40 °C; 140 µs; 97 kJ/kg, exit temperature: 45 °C; 168 µs, 116,7 kJ/kg; exit temperature: 50 °C; 173 µs, 116.7 kJ/kg; exit temperature: 50 °C.

Several authors have reported that when PEF treatments are applied to a microbial population, a proportion of cells is sublethally injured, and this is more evident when moderate conditions are applied [36]. The final recovery or death of that injured population is directly dependent on either optimal or adverse recovery conditions [37,38]. Preventing the reparation of sublethal injuries caused by PEF by adding preservatives is a strategy that can increase the lethality of moderate PEF treatments [39,40,41,42]. Figure 2 illustrates the inactivation of *S. cerevisiae* (Figure 2A) and *O. oeni* (Figure 2B) in the respective wines after fermentation by combining PEF treatments of different durations at 15 kV/cm with the addition of 20 ppm of SO_2_ evaluated 24 h after processing. The lethal effects of the individual treatments are also shown in order to identify if the effect of the combined treatments was additive or synergic. Figure 2A,B show that the added SO_2_ scarcely affected the viability of the untreated cells of *S. cerevisiae* and *O. oeni*. On the other hand, the inactivation of PEF-treated cells of *S. cerevisiae* maintained for 24 h in wine without added SO_2_ increased significantly. For example, the inactivation detected just after the 140 µs PEF treatment increased from 2.0 to 4.0 Log_10_ cycles after 24 h. This effect could be explained by the supposition that the presence of ethanol and the content of free sulfites in the wine (14.7%; 19.2 ppm, see Table 1) prevented the recovery of a proportion of the yeast cells that had been sublethally injured as a consequence of the PEF treatment. The addition of 20 ppm of SO_2_ barely increased lethality compared with wines treated solely with PEF; the difference is not statistically significant (*p* > 0.05). The yeast cells that survived in PEF-treated wine during 24 h were thus unaffected by the addition of 20 ppm of extra sulfites.

The incubation of PEF-treated *O. oeni* cells for 24 h in wine without added SO_2_ did not significantly increase the lethality of the treatments applied over different time intervals. This observation confirms results obtained by other authors who reported that sublethal injury did not occur when Gram-positive bacteria, such as *O. oeni*, were treated by PEF in media of low pH [43,44]. Regarding the combination of PEF with SO_2_, the most effective combination was observed 24 h after mixing 20 ppm of SO_2_ with wine treated for 173 µs. The PEF treatment thus sensitized a proportion of the surviving population to SO_2_, whereby the lethality of the combined treatment was over 2.0 Log_10_ cycles more than the sum of the single treatments. The main mechanism involved in the antimicrobial effect of SO_2_ on yeast is related to the diffusion of SO_2_ into the cytoplasm and the subsequent disturbance of metabolic processes by the binding of SO_2_ to essential molecules (proteins, nucleic acids, coenzymes, cofactors, vitamins, etc.). Although the activity of SO_2_ against bacteria is still unclear, the electroporation phenomenon triggered by PEF could enhance the diffusion of SO_2_ into the cytoplasm of *O. oeni*, thereby exerting an effect similar to the one described for yeast [4].

### 3.3. Evolution of the Microbial Population in Wine Treated by PEF after Alcoholic and Malolactic Fermentation during 4 Months of Storage

Based on preliminary results obtained on the resistance of *S. cerevisiae* and *O. oeni* to PEF treatments of different intensities and their combination with SO_2_, treatment conditions with the aim of evaluating the evolution of the microbial population in wines during 4 months of storage were chosen.

Treatments at 15 kV/cm of a shorter (62 µs) and longer (140 µs) duration that corresponded to a total specific energy of 39 and 97.2 kJ/kg and an outlet temperature of 30 and 45 °C, respectively, were selected for wine obtained after alcoholic fermentation. Table 2 shows the evolution of yeast populations in wine treated by the two selected PEF treatments after alcoholic fermentation without adding SO_2_ or with the addition of 20 ppm of SO_2_. For comparative purposes, the evolution of the yeast populations in the untreated wines with and without 20 ppm of added SO_2_ is also included. The initial number of viable yeast cells in the untreated wines was 3 × 10^6^ CFU/mL. This initial number was maintained during the first month of storage, even in the untreated wine dosed with 20 ppm of sulfites. After 4 months of storage, the yeast cell population decreased by about 3.0 logarithmic units. These results indicate that the addition of 20 ppm of SO_2_ did not compromise the viability of yeast cells that fermented a wine which already had 19 ppm of free SO_2_ by the end of fermentation. The reduction of the yeast cell population observed between 1 and 4 months might be the loss of viability that occurs in a microbial population when it is maintained under conditions in which multiplication does not occur. In the case of PEF-treated wines, a similar reduction in yeast viability as in the control wine after 4 months was achieved just after the application of the most intense PEF_2_ treatment (97.2 kJ/kg; 171 µs), or after 1 day of incubation in the wine treated at lower intensity PEF_1_ (39 kJ/kg; 83 µs). After 4 months of incubation, no viable yeasts were detected in the two wines, even when SO_2_ was not added to them.

In the case of the wine that had undergone malolactic fermentation, as sublethal injury was not detected in *O. oeni* cells after PEF treatment, the two longer treatments (132 and 173 µs) that correspond to a total specific energy of 77.8 and 116.7 kJ/kg, respectively (outlet temperatures of 40 and 50 °C), were selected. Moreover, in order to obtain synergetic effects, the low-intensity treatment (PEF_3_) was combined with the addition of 30 ppm of sulfites, and the high-intensity treatment (PEF_4_) was combined with the addition of 20 ppm of sulfites.

As the presence of yeast in addition to *O. oeni* was detected in the wine obtained after malolactic fermentation, the evolution of the two microorganisms under the different assayed conditions is shown in Table 3. It shows that the initial concentration of *O. oeni* after malolactic fermentation (≈10^5^ CFU/mL) slowly decreased during storage in untreated wines with no added SO_2_, as well as in those with 20 or 30 ppm of SO_2_ added; the decrease was slightly higher in the latter ones. After 4 months of storage, the differences in concentration of viable cells between the untreated wine without added SO_2_ and with 30 ppm added was lower than 1.0 Log_10_ cycle. On the other hand, as had been observed in the case of the yeast after alcoholic fermentation, the population of *O. oeni* decreased more rapidly in wines treated by PEF. After 4 months of storage, the concentration of viable cells was around 2 × 10^3^ and 6 × 10^2^ CFU/mL in wines without added SO_2_ but treated with low and high intensity PEF, respectively. However, in wines with 20 or 30 ppm of SO_2_, the number of viable cells of *O. oeni* was lower than 1 × 10^2^ CFU/mL, independently of the intensity of the PEF treatment applied. It is remarkable that in both PEF-treated wines combined with added SO_2_, a pronounced synergetic effect could be observed, obtaining a reduction of 2.0–3.0 Log_10_ cycles of the initial *O. oeni* population within just 24 h.

Regarding the evolution of yeast, Table 3 shows that the concentration of yeast populations in wine after malolactic fermentation was around 0.5 log cycles lower than in wine after alcoholic fermentation (Table 2). This confirms that yeast viability after alcoholic fermentation decreases along time. Untreated yeast populations in wine after malolactic fermentation decreased along storage time: the number of viable yeasts after 4 month of storage was less than 1 × 10^2^ CFU/mL. As occurred in the case of wine after alcoholic fermentation, the addition of 20 ppm of SO_2_ did not significantly affect the yeasts’ viability along storage as compared to the wine without added SO_2_ that contained 10 ppm of free SO_2_ at the moment of the trial. After 15 days of incubation, viable yeast cells were not detected in the wines treated by PEF at the two assayed intensities, independently of whether SO_2_ had been added or not.

These results thus evidence the capacity of PEF, even applied at quite moderate intensities, as a potential alternative alone or in combination with sulfites, for microbial control at different steps of the red wine production process. The inactivation of yeast by means of PEF after alcoholic fermentation would allow for the achievement of a lower free SO_2_ content, thereby facilitating the implementation of LAB for further malolactic fermentation. González-Arenzana et al., (2018) [35] reported a shortening of the malolactic fermentation time for wines pre-treated by PEF, attributing this reduction of fermentation time to the decrease in competitive pressure for lactic acid bacteria. Furthermore, the complete decontamination of yeasts after alcoholic fermentation would be of special interest for sweet or semi-sweet wines with residual sugars, in order to prevent re-fermentation [45]. On the other hand, in addition to preventing re-fermentation by yeast, PEF alone, or in combination with SO_2_ after malolactic fermentation, could contribute to the prevention spoilage caused by lactic acid bacteria during storage in bottles, which generally leads to phenomena such as a slimy appearance, undesirable off-flavors, and/or the production of biogenic amines [3].

### 3.4. Effect of PEF Treatments on the Oenological Parameters of Wine after 4 Months of Storage

Any new technique that might be potentially introduced in wineries should guarantee zero drawbacks in terms of physicochemical parameters of wine. Furthermore, as a potential alternative to SO_2_, which acts as an antioxidant, PEF technology should not trigger oxidative reactions that compromise wine quality. Thus, once the effectivity of PEF treatments for controlling different microbial populations along the winemaking process had been observed, the effect of PEF on the oenological parameters of wines was evaluated. After 4 months of storage, the oenological parameters of the untreated and PEF-treated wines, with and without the addition of SO_2_ after alcoholic and malolactic fermentation, are shown in Table 4 and Table 5, respectively.

The results in Table 4 and Table 5 show no significant differences between the PEF-treated wines and the untreated wines from a practical point of view in terms of pH, glucose-fructose, % ethanol, and total and volatile acidity. Neither did PEF treatments significantly affect indexes related to polyphenol content, such as color index, total polyphenol index, and total anthocyanin content. These results support previous studies which reported that PEF treatments applied at moderate intensities did not impair wine properties [20,46].

### 3.5. Evaluation of the Effect of PEF Versus the Sterilizing Filtration Method on the Oenological and Sensory Properties of a Commercial Red Wine

Red wine is a valuable product in which, in addition to physicochemical properties, it is especially vital to maintain sensory characteristics. Color, flavor, and aroma characteristics are essential in wine quality. In order to prevent undesirable effects on the sensory properties of wine, thermal treatments have traditionally been avoided in the wine industry for microbial decontamination in comparison with other food industries. Consequently, before bottling, wine frequently undergoes a sterilizing filtration treatment to avoid microbial spoilage during its subsequent shelf life.

As pointed out above, when a liquid food is processed by PEF in a continuous flow, a temperature increment in the food occurs because all of the electrical energy required to generate the electric field is transformed into heat. In order to ascertain whether that temperature increment affected the oenological and sensory properties of wine, we compared a commercial wine ready for bottling after sterilizing filtration with the same wine treated by PEF at two different intensities that corresponded to outlet temperatures of 40 and 60 °C. The oenological parameters of wines either untreated, PEF-treated, or sterilized by filtration after 1 month of storage are presented in Table 6. In comparison to the untreated wine, PEF treatments did not affect oenological parameters, even in the case of the more intense treatment applied (155.6 kJ/kg; 60 °C). In contrast, the only difference found in Table 6 corresponds with the color index (CI) of the wine treated by means of sterilizing filtration, which was significantly lower (*p* ≤ 0.05). This finding confirms that microbial sterilization by means of a filtration process is a harsh procedure that affects wine color [47,48].

Regarding the effect of PEF on sensory properties of wine, Table 7 shows the results of the triangle test comparing the aroma and taste of wines sterilized by filtration and treated by PEF at two intensities. Results shown in Table 7 indicate that panelists were not able to detect differences in aroma and taste between wine after sterilizing filtration and the PEF-treated wines, even those treated at the highest intensity. More than 56% of correct responses are required for differences to be considered statistically significant (*p* < 0.05) [24].

Therefore, results obtained in the sensory study demonstrated that the rapid, brief temperature increment that occurred in a red wine as a consequence of the application of a PEF treatment did not negatively impair its oenological and sensory properties, even when the wine’s outlet temperature was 60 °C. This result is particularly relevant and underscores PEF’s potential for implementation as a microbial control method in the wine industry.

## 4. Conclusions

This study reinforces the growing evidence that PEF is a technology that can be potentially successful in helping to reduce the number of sulfites used in wineries, not only due to its effectivity in inactivating different microorganisms, but also due to its zero detrimental effect on wine quality.

## Figures and Tables

**Table 1:** Initial oenological parameters of red wines after alcoholic (AF) and malolactic fermentation (MLF).

	Alcoholic Fermented (AF) Red Wine	Malolactic Fermented (MLF) Red Wine
pH	3.80 ± 0.01	3.54 ± 0.01
Glucose-Fructose (g/L)	0.94 ± 0.02	0.29 ± 0.02
% Ethanol (*v*/*v*)	14.70 ± 0.04	14.35 ± 0.03
Total Acidity (g/L) ^a^	4.34 ± 0.32	5.17 ± 0.14
Volatile Acidity (g/L) ^b^	0.59 ± 0.03	0.45 ± 0.02
Free SO_2_ (ppm) ^c^	19.2 ± 3.2	10.0 ± 3.2
Total SO_2_ (ppm) ^c^	22.4 ± 3.2	16.0 ± 3.2
CI * (A.U.)	20.48 ± 0.36	13.07 ± 0.41
TPI ** (A.U.) ^d^	67.3 ± 0.14	54.62 ± 0.52
TAC *** (mg/L) ^e^	982.09 ± 8.05	532.21 ± 7.39

Values represent mean with standard deviation. * Color intensity. ** Total polyphenol index. *** Total anthocyanin content. A.U.: absorbance units. ^a^: expressed as tartaric acid. ^b^: expressed as acetic acid. ^c^: expressed as the mean ± the deviation of the analytical method. ^d^: expressed as tartaric acid. ^e^: expressed as malvidin-3-glucoside.

**Table 2:** Evolution of Log_10_ CFU/mL of *S. cerevisiae* cells during the storage time of red wine after alcoholic fermentation (AF) treated by PEF and added with different SO_2_ concentrations (0 or 20 ppm). PEF1 (15 kV/cm, 39 kJ/kg, exit temperature: 30 °C) and PEF2 (15 kV/cm, 97.2 kJ/kg, exit temperature 45 °C).

	0 Days	1 Day	7 Days	15 Days	1 Month	4 Months
Control	6.46 ± 0.04 *a*	6.47 ± 0.03 *a*	6.21 ± 0.05 *a*	6.28 ± 0.21 *a*	5.93 ± 0.06 *a*	3.69 ± 0.04 *a*
Control 20 ppm SO_2_	6.40 ± 0.02 *a*	6.46 ± 0.04 *a*	6.20 ± 0.06 *a*	6.18 ± 0.12 *a*	5.97 ± 0.10 *a*	3.93 ± 0.15 *b*
PEF_1_	5.94 ± 0.04 *b*	3.39 ± 0.01 *b*	3.24 ± 0.10 *b*	3.24 ± 0.04 *b*	3.10 ± 0.01 *b*	n.d. *c*
PEF_1_ 20 ppm SO_2_	5.89 ± 0.01 *b*	3.12 ± 0.03 *c*	3.08 ± 0.02 *c*	3.25 ± 0.11 *b*	2.32 ± 0.76 *bc*	n.d. *c*
PEF_2_	2.36 ± 0.04 *c*	2.35 ± 0.04 *d*	2.31 ± 0.01 *d*	2.34 ± 0.08 *c*	2.38 ± 0.04 *bc*	n.d. *c*
PEF_2_ 20 ppm SO_2_	2.79 ± 0.08 *d*	3.03 ± 0.11 *c*	2.96 ± 0.03 c	2.39 ± 0.03 *c*	2.19 ± 0.01 *c*	n.d. *c*

Values represent mean with standard deviation. Different letters within the same column indicate significant differences (*p* ≤ 0.05). n.d. = not detected. <1.5 Log_10_ CFU/mL = below the quantification limit (30 CFU/mL).

**Table 3:** Evolution of the Log_10_ CFU/mL of O. oeni cells and yeast cells during the storage time of wine after malolactic fermentation (MLF) treated by PEF and added with different SO_2_ concentrations (0, 20 or 30 ppm). PEF3 (15 kV/cm, 77.8 kJ/kg, exit temperature: 40 °C) and PEF4 (15 kV/cm, 116.7 kJ/kg, exit temperature: 50 °C).

	0 Days		1 Day		15 Days		1 Month		4 Month	
	*O. oeni*	Yeast	*O. oeni*	Yeast	*O. oeni*	Yeast	*O. oeni*	Yeast	*O. oeni*	Yeast
Control	5.14 ± 0.08 *a*	5.97 ± 0.08 *a*	4.98 ± 0.01 *a*	5.94 ± 0.02 *a*	4.64 ± 0.08 *a*	3.43 ± 0.01 *a*	4.42 ± 0.04 *a*	2.79 ± 0.21 *a*	4.31 ± 0.08 *a*	1.51 ± 0.03 *ab*
Control 20 ppm SO_2_	5.13 ± 0.04 *a*	5.98 ±0.09 *a*	4.52 ± 0.06 *b*	4.55 ± 0.04 *b*	4.04 ± 0.05 *a*	3.10 ± 0.01 *b*	4.43 ± 0.02 *a*	1.99 ± 0.11 *b*	3.95 ± 0.19 *ab*	1.43 ± 0.07 *a*
Control 30 ppm SO_2_	5.10 ± 0.06 *a*	5.96 ± 0.08 *a*	4.08 ± 0.01 *c*	3.43 ± 0.04 *c*	2.98 ± 0.14 *b*	3.04 ± 0.27 *b*	2.80 ± 0.20 *b*	1.80 ± 0.01 *b*	3.62 ± 0.06 *bc*	1.75 ± 0.23 *b*
PEF_3_	4.50 ± 0.01 *b*	2.02 ± 0.03 *b*	4.43 ± 0.01 *b*	1.78 ± 0.11 *d*	4.39 ± 0.54 *a*	0.80 ± 0.14 *c*	4.48 ± 0.06 *a*	n.d. *c*	3.37 ± 0.06 *c*	n.d. *c*
PEF_3_ 30 ppm SO_2_	4.49 ± 0.01 *b*	1.94 ± 0.13 *b*	2.90 ± 0.07 *d*	<1.5 *e*	2.35 ± 0.49 *bc*	n.d. *d*	1.54 ± 0.22 *c*	n.d. *c*	1.73 ± 0.31 *d*	n.d. *c*
PEF_4_	3.57 ± 0.04 *c*	1.89 ± 0.16 *b*	2.15 ± 0.18 *e*	<1.5 *e*	2.55 ± 0.10 *b*	n.d. *d*	3.61 ± 0.17 *d*	n.d. *c*	2.82 ± 0.25 *e*	n.d. *c*
PEF_4_ 20 ppm SO_2_	3.55 ± 0.05 *c*	1.83 ± 0.06 *b*	<1.5 *f*	<1.5 *e*	1.73 ± 0.02 *c*	n.d. *d*	1.59 ± 0.16 *c*	n.d. *c*	1.90 ±0.23 *d*	n.d. *c*

Values represent mean with standard deviation. Different letters within the same column indicate significant differences (*p* ≤ 0.05). n.d. = not detected. <1.5 Log_10_ CFU/mL = below the quantification limit (30 CFU/mL).

**Table 4:** Oenological parameters after 4 months of storage of red wine after alcoholic fermentation (AF) treated by PEF and added with different SO_2_ concentrations (0 or 20 ppm). PEF1 (15 kV/cm, 39 kJ/kg, exit temperature: 30 °C) and PEF2 (15 kV/cm, 97.2 kJ/kg, exit temperature 45 °C).

	Control		PEF_1_		PEF_2_	
	SO_2_	SO_2_	SO_2_	SO_2_	SO_2_	SO_2_
	0 ppm	20 ppm	0 ppm	20 ppm	0 ppm	20 ppm
pH	3.81 ± 0.02	3.80 ± 0.01	3.80 ± 0.00	3.82 ± 0.02	3.77 ± 0.02	3.80 ± 0.01
Glucose-Fructose (g/L)	0.92 ± 0.01	0.96 ± 0.02	1.33 ± 0.03	1.41 ± 0.03	1.31 ± 0.02	1.35 ± 0.02
% Ethanol (*v*/*v*)	14.74 ±0.15	14.71 ± 0.04	14.69 ± 0.07	14.70 ± 0.03	14.72 ± 0.03	14.70 ± 0.02
Total Acidity (g/L) ^a^	4.00 ± 0.22	4.40 ± 0.19	4.55 ± 0.10	4.40 ± 0.17	4.48 ± 0.22	4.48 ± 0.08
Volatile Acidity (g/L) ^b^	0.61 ± 0.03	0.56 ± 0.03	0.63 ± 0.01	0.57 ± 0.03	0.58 ± 0.06	0.56 ± 0.02
Free SO_2_ (ppm) ^c^	9.6 ± 3.2	12.8 ± 3.2	9.6 ± 3.2	12.8 ± 3.2	9.6 ± 3.2	12.8 ± 3.2
Total SO_2_ (ppm) ^c^	22.4 ± 3.2 a	32 ± 3.2 b	22.4 ± 3.2 a	32 ± 3.2 b	22.4 ± 3.2 a	32 ± 3.2 b
CI * (A.U.)	16.17 ± 0.23	16.45 ± 1.18	17.77 ± 0.62	16.59 ± 0.49	17.33 ± 0.00	18.22 ± 1.00
TPI ** (A.U.) ^d^	60.75 ± 1.48	61.10 ± 0.42	61.10 ± 0.85	60.65 ± 0.49	60.05 ± 0.07	60.60 ± 0.14
TAC *** (mg/L) ^e^	645.25 ± 11.27 a	691.81 ± 9.18 b	633.87 ± 1.61 a	683.28 ± 11.36 b	636.14 ± 1.61 a	691.80 ± 10.68 b

Values represent mean with standard deviation. Different letters within the same row indicate significant differences (*p* ≤ 0.05). * Color intensity. ** Total polyphenol index. *** Total anthocyanin content. A.U.: absorbance units. ^a^: expressed as tartaric acid. ^b^: expressed as acetic acid. ^c^: expressed as the mean ± the deviation of the analytical method. ^d^: expressed as tartaric acid. ^e^: expressed as malvidin-3-glucoside.

**Table 5:** Oenological parameters after 4 months of storage of red wine after malolactic fermentation (MLF) treated by PEF and added with different SO_2_ concentrations (0, 20 or 30 ppm). PEF3 (15 kV/cm, 77.8 kJ/kg, exit temperature: 40 °C) and PEF4 (15 kV/cm, 116.7 kJ/kg, exit temperature: 50 °C).

	Control			PEF_3_		PEF_4_	
	SO_2_	SO_2_	SO_2_	SO_2_	SO_2_	SO_2_	SO_2_
	0 ppm	20 ppm	30 ppm	0 ppm	30 ppm	0 ppm	20 ppm
pH	3.54 ± 0.01	3.54 ± 0.01	3.54 ± 0.01	3.53 ± 0.00	3.52 ± 0.01	3.51 ± 0.01	3.52 ± 0.01
Glucose-Fructose (g/L)	0.27 ± 0.02	0.29 ± 0.02	0.30 ± 0.02	0.27 ± 0.03	0.30 ± 0.02	0.27 ± 0.02	0.29 ± 0.02
% Ethanol (*v*/*v*)	14.39 ± 0.05	14.35 ± 0.03	14.33 ± 0.04	14.37 ± 0.01	14.30 ± 0.04	14.32 ± 0.02	14.29 ± 0.04
Total Acidity (g/L) ^a^	5.37 ± 0.14	5.15 ± 0.12	5.07 ± 0.14	5.22 ± 0.09	5.07 ± 0.07	5.15 ± 0.05	5.15 ± 0.00
Volatile Acidity (g/L) ^b^	0.53 ± 0.02	0.44 ± 0.02	0.42 ± 0.04	0.48 ± 0.04	0.42 ± 0.02	0.48 ± 0.05	0.42 ± 0.02
Free SO_2_ (ppm) ^c^	6.4 ± 3.2	12.8 ± 3.2	12.8 ± 3.2	9.6 ± 3.2	12.8 ± 3.2	6.4 ± 3.2	12.8 ± 3.2
Total SO_2_ (ppm) ^c^	16 ± 3.2 a	22.4 ± 3.2 ab	25.6 ± 3.2 b	19.2 ± 3.2 ab	25.6 ± 3.2 b	16 ± 3.2 a	22.4 ± 3.2 ab
CI * (A.U.)	12.80 ± 0.12	12.16 ± 0.03	12.35 ± 0.18	13.51 ± 1.19	13.24 ± 0.28	13.40 ± 0.47	13.87 ± 0.22
TPI ** (A.U.) ^d^	50.07 ± 2.11	50.25 ± 1.07	49.92 ± 0.80	50.89 ± 3.84	50.00 ± 0.40	49.63 ± 1.43	49.08 ± 0.03
TAC *** (mg/L) ^e^	327.69 ± 10.41	345.12 ± 19.97	345.46 ± 11.41	309.72 ± 11.88	306.33 ± 22.89	314.95 ± 7.76	323.52 ± 8.16

Values represent mean with standard deviation. Different letters within the same row indicate significant differences (*p* ≤ 0.05). * Color intensity. ** Total polyphenol index. *** Total anthocyanin Content. A.U.: absorbance units. ^a^: expressed as tartaric acid. ^b^: expressed as acetic acid. ^c^: expressed as the mean ± the deviation of the analytical method. ^d^: expressed as tartaric acid. ^e^: expressed as malvidin-3-glucoside.

**Table 6:** Oenological parameters after 1 month of storage of untreated, PEF-treated, or sterilizing filtrated red wine. PEF treatments: PEF_A_ (15 kV/cm, 195 µs, 84.5 kJ/kg exit temperature: 40 °C) or PEF_B_ (15 kV/cm, 310 µs, 155.6 kJ/kg, exit temperature: 60 °C).

	Untreated	PEF_A_ 84.5 kJ/kg; 40 °C	PEF_B_ 155.6 kJ/kg; 60 °C	Sterilizing Filtration
pH	3.54 ± 0.05	3.53 ± 0.02	3.53 ± 0.04	3.53 ± 0.02
% Ethanol (*v*/*v*)	13.73 ± 0.12	13.75 ± 0.20	13.76 ± 0.14	13.70 ± 0.12
Total Acidity (g/L) ^a^	4.85 ± 0.19	4.70 ± 0.22	4.70 ± 0.18	4.80 ± 0.22
Volatile Acidity (g/L) ^b^	0.49 ± 0.02	0.49 ± 0.01	0.49 ± 0.01	0.49 ± 0.02
Malic Acid (g/L)	0.07 ± 0.01	0.08 ± 0.01	0.08 ± 0.02	0.07 ± 0.03
Free SO2 (ppm) ^c^	32.0 ± 3.2	32.0 ± 4.2	35.2 ± 5.3	36.0 ± 3.2
Total SO2 (ppm) ^c^	80.0 ± 7.2	80.0 ± 3.2	80.0 ± 5.24	80.0 ± 3.1
CI * (A.U.)	11.6 ± 1.4 a	11.3 ± 0.2 a	12.9 ± 0.8 a	8.0 ± 1.2 b
TPI ** (A.U.) ^d^	59.3 ± 1.4	59.8 ± 1.2	60.2 ± 1.0	57.9 ± 1.2
TAC *** (mg/L) ^e^	291.4 ± 5.1	282.3 ± 10.1	282.9 ± 12.4	284.8 ± 14.1

Values represent mean with standard deviation. Different letters within the same row indicate significant differences (*p* ≤ 0.05). * Color Intensity. ** Total Polyphenol Index. *** Total Anthocyanin Content. A.U.: absorbance units. ^a^: expressed as tartaric acid. ^b^: expressed as acetic acid. ^c^: expressed as the mean ± the deviation of the analytical method. ^d^: expressed as tartaric acid. ^e^: expressed as malvidin-3-glucoside.

**Table 7:** Percentage of correct responses in a triangle test comparing untreated, PEF-treated, and sterilizing filtrated red wine after 1 month of storage. PEF treatments: PEF_A_ (15 kV/cm, 195 µs, 84.5 kJ/kg exit temperature: 40 °C) or PEF_B_ (15 kV/cm, 310 µs, 155.6 kJ/kg, exit temperature: 60 °C).

Triangle Test (Percentage of Correct Responses)	
PEF_A_/PEF_B_	50.0%
PEF_A_/Sterilizing Filtration	22.2%
PEF_B_/PEF_B_	33.3%
PEF_B_/Sterilizing Filtration	38.9%
Sterilizing Filtration/PEF_A_	50.0%
Sterilizing Filtration/PEF_B_	44.4%

## Data Availability

Data is contained within the article.
